# Study of Bond–Slip Behavior and Constitutive Model of a New M-Section Steel-Skeleton Concrete

**DOI:** 10.3390/ma15196776

**Published:** 2022-09-29

**Authors:** Jun Wei, Qingshun Yang, Yang Yu, Qing Wang, Lexiang Zhou, Fei Chen

**Affiliations:** 1 College of Civil Engineering, Qinghai University, Xining 810016, China; 2 Qinghai Provincial Key Laboratory of Energy-Saving Building Materials and Engineering Safety, Xining 810016, China; 3 Centre for Infrastructure Engineering and Safety (CIES), School of Civil and Environmental Engineering, The University of New South Wales, Sydney, NSW 2052, Australia; 4 Beijing Zhongqing Hengye Technology Development Co., Beijing 100044, China

**Keywords:** push-out test, M-section steel–concrete, DIC-3D, bond–slip constitutive model, numerical simulation

## Abstract

In this study, the bond–slip behavior between a new type of M-section steel skeleton (i.e., M-section steel) applied in assembled shear wall structures and concrete was investigated. First, push-out tests were conducted on 21 M-section steel–concrete (MSSC) specimens, wherein the effects of the concrete cover, concrete strength, and anchorage length on the bond strength between the M-section steel and concrete were considered. Further, the crack patterns, strain distribution of M-section steel, and bond–slip curves of the MSSC specimens were investigated using conventional strain measurement and a non-contact optical three-dimensional deformation measurement system, Digital Image Correlation-3D (DIC-3D). The experimental results demonstrated that the bond–slip curves of the MSSC specimens were divided into four stages: the linear ascending, non-linear ascending, non-linear descending, and residual stages. The initial average bond strength ${\bar{\tau }}_{s}$ was mainly affected by the concrete strength and anchorage length, whereas the concrete cover and anchorage length influenced the residual average bond strength ${\bar{\tau }}_{r}$, and the ultimate average bond strength ${\bar{\tau }}_{u}$ was affected by the concrete strength, concrete cover, and anchorage length. Consequently, a bond–slip constitutive model of M-section steel and concrete was proposed based on the experimental results, and consistency was observed in comparison with the test results, which verified the applicability of the proposed model. Furthermore, to verify the rationality of the bond–slip constitutive model, a numerical simulation was performed, wherein the bond–slip curves, stress clouds, and interfacial bond damage process of the MSSC specimens were investigated. The numerical simulation results indicated that the bond–slip constitutive model could accurately predict the entire failure process of the MSSC specimens.

## 1. Introduction

With the advances in building industrialization, assembled lightweight steel forming skeletons have been applied in shear wall structures due to their characteristics of high stress-efficiency, seismic energy-efficiency, and environmentally friendly protection [1,2,3]. However, Xu et al. [4] observed that the bond–slip phenomenon occurred in the process of low-cycle repeated tests of lightweight steel–concrete shear walls. The concrete and lightweight steel were separated from each other and could not work together as a whole, which directly affected the mechanical performance, damage pattern, bearing capacity, and deformation of all the components.

**Figure 1:**
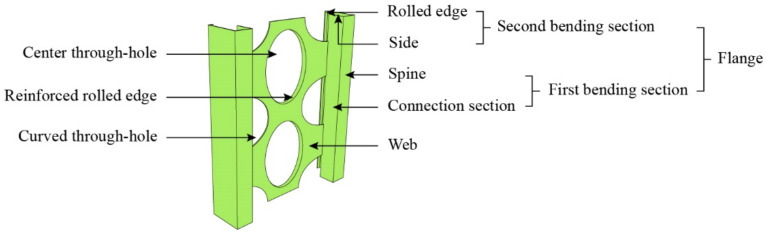
M-section steel skeleton.

Chen et al. [5] considered that the shape of the steel section has a large effect on its bond strength upon studying the bond–slip performance between checkered steel and concrete. Therefore, to enhance the bond strength between the lightweight steel forming skeleton and concrete and improve the advantages (i.e., the load-bearing capacity, energy dissipation performance, etc.) of the whole structure, a new type of M-section steel forming skeleton (i.e., M-section steel) was proposed [6,7], as shown in Figure 1. The main structure of M-section steel is composed of a web and flanges, and is used to replace the original longitudinal reinforcement. Curved through-holes are opened through the webs, mainly for the arrangement of horizontal reinforcement. However, the bond–slip performance of M-section steel in concrete is quite different from the currently studied steel bar in concrete [8,9,10,11,12,13], steel-reinforced concrete [14,15,16], steel-reinforced recycled concrete [17,18,19,20,21,22,23,24,25], steel-reinforced engineered cementitious composites [26,27,28], and steel plate in concrete [29,30,31,32,33]. Therefore, to achieve the popularization of M-section steel-skeleton shear walls, it is very necessary to carry out further research on the interfacial bonding behavior between M-section steel and concrete.

To study the bond strength between M-section steel and concrete, 21 push-out specimens were analyzed. The effects of the concrete cover, concrete strength, and anchorage length on the bond strength between the M-section steel and concrete were considered. Thereafter, based on the experimental results, a bond–slip constitutive model between the M-section steel and concrete was proposed. A numerical simulation was conducted using the proposed constitutive equation, which demonstrates that the proposed equation better predicted the damage process of the entire specimen.

## 2. Experimental Design

### 2.1. Specimen Design

Liu et al. [21,22,23] concluded that the concrete strength ($f_{cu}$), concrete cover ($C_{ss}$) (i.e., the distance from the concrete surface to the surface of the reinforced steel, denoted by $C_{ss}$), and anchorage length ($L_{e}$) have a large effect on the bond strength between steel-reinforced and concrete.

**Figure 2:**
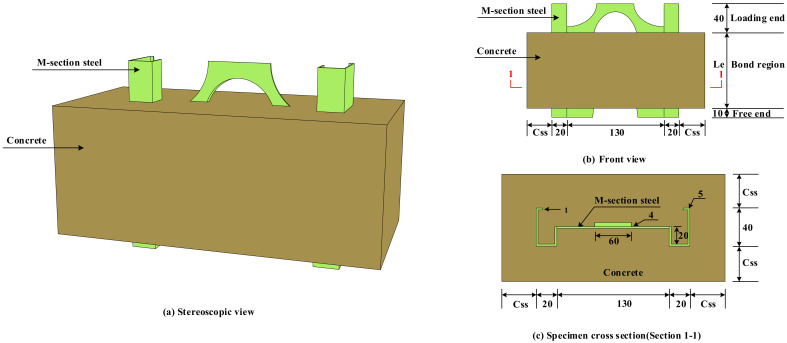
Schematic of MSSC specimens (unit: mm).

**Figure 3:**
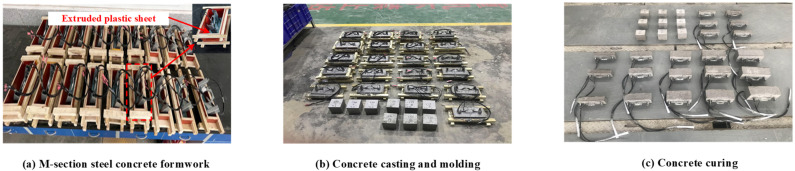
Preparation process of MSSC specimens.

Therefore, the influence of concrete strength ($f_{cu}$), concrete cover ($C_{ss}$), and anchorage length ($L_{e}$) on the bond–slip performance of M-section steel and concrete was mainly considered in this test. We designed and produced 7 groups of specimens to launch the test, setting three specimens in each to reduce the error caused by data dispersion. The specimen parameters are presented in Table 1. Figure 2 presents the three-dimensional elevation of the launched specimen and the detailed dimensions of each part. A size of 10 mm × 40 mm × 20 mm × 20 mm × 130 mm × 1 mm was used for all the M-section steel cross-sectional dimensions, as illustrated in Figure 2c. Figure 3 shows the details of the fabrication process of the MSSC specimens, wherein the concrete of the specimens was cast horizontally. The free-end length was preserved by setting an extruded plastic sheet with a thickness of 10 mm at the bottom of the M-section steel, and the cross-sectional dimensions of the extruded plastic sheet varied with the size of the formwork, as shown in Figure 3a.

### 2.2. Material Properties

**Figure 4:**
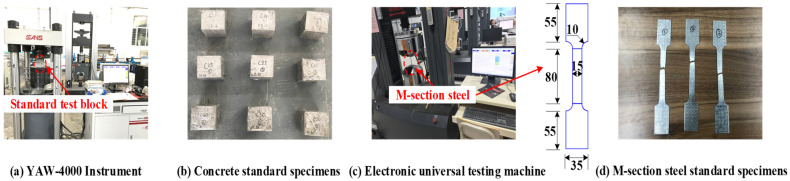
Material test.

The compressive strengths of standard cubes of concrete used in the test were 20 MPa, 25 MPa, and 30 MPa, respectively [34,35]. The YAW-4000 microcomputer-controlled electro-hydraulic servo pressure testing machine (MTS Systems (China) Co., Ltd., Guangdong, China) at Qinghai University (Xining, China) was used for the cube axial compressive strength test of the concrete, as shown in Figure 4a,b. Table 2 shows the basic properties of the concrete. The M-section steel was made of Q235 steel, and a standard specimen, as shown in Figure 4c, was fabricated using the room-temperature tensile test method for metallic materials (GB/T228-2010) [36]. Each segment of steel was machined as a non-proportional specimen with a rectangular cross-section (three in total). Further, the ETM305D electro-hydraulic servo machine (Shenzhen WANCE Testing Machine Co., Ltd, Tangwei, China) at Qinghai University was employed to perform the M-section steel property test at a loading rate of 0.5 mm/min. The specimen was abruptly pulled off at the test end according to the expected pull-off position with the fracture surface at an approximate angle of 30° to the length of the specimen. The final failure mode is illustrated in Figure 4d. The basic parameters of the M-section steel based on the test results are listed in Table 3.

### 2.3. Loading and Measurement Schemes

**Figure 5:**
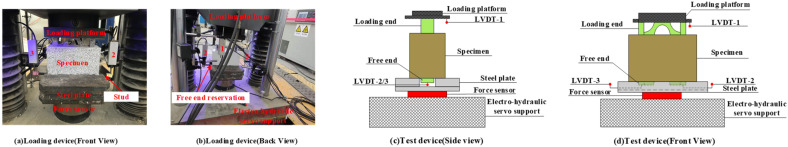
Push-out test device diagram.

The ETM305D electro-hydraulic servo universal testing machine at Qinghai University was used for the monotonic loading test of the MSSC specimens, as shown in Figure 5. In the loading device shown in the figure, to consider the test accuracy, a 300 KN force transducer was erected at the bottom of the steel plate for load collection. Simultaneously, the upper bearing platform of the universal testing machine was used as the active force transmission plate, whereas the lower one was used as the fixed force transmission plate. For M-section steel, the upper and lower ends are the loading and free ends, respectively. The steel plates were placed above the force sensor at the free end of the reservation, as shown in Figure 5c. The steel plates comprised two pieces of 300 mm × 300 mm × 10 mm and four pieces of 100 mm × 300 mm × 20 mm Q345 steel welded together, and the pre-drilled holes are shown in Figure 5b. During the test, the surface of the protruding M-section steel at the loading end was polished to ensure uniform force on the specimen throughout the loading process and to avoid local buckling of the protruding M-section steel owing to accidental eccentricity and other factors. In addition, to prevent diagonal compression damage at the bottom of the concrete during the test and ensure that only bond–slip occured, the steel plates were placed close to the M-section steel.

This test was performed using displacement-controlled loading at a loading rate of 0.3 mm/min. When the displacement at the loading end reached approximately 3–5 mm or a significant crack appeared in the concrete during the loading process, the loading ended. Because the M-section steel was only subjected to the force of the upper bearing and the bonding force to the concrete, the force sensor readings can be used as the bonding force examined in this test. A magnetic meter holder was used at the loading end to fix the linear variable differential transformer (LVDT) at the upper bearing of the test machine, which was used to the measure the relative slip at the loading end, as shown for LVDT-1 in Figure 5d. Two bolts are welded to the free end of the M-section steel, and then, the roots of the bolts were clamped using a dovetail clip such that the LVDT could be fixed and moved together with the bolts. The slip of the bolts is the relative slip between the free end of the M-section steel and the concrete, as shown in Figure 5a. To ensure the accuracy of the test, the relative slip of the free end on both sides was collected for comparative analysis, as illustrated in Figure 5d for LVDT-2 and LVDT-3.

**Figure 6:**
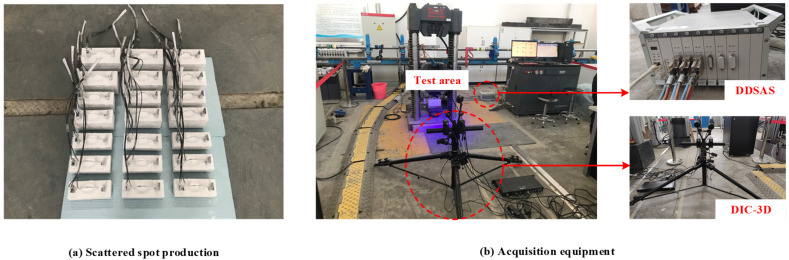
Acquisition layout diagram.

The LVDT, strain gauges, and force transducer data were obtained using the Donghua dynamic signal acquisition system (DDSAS) [22] at Qinghai University. In addition, a non-contact optical three-dimensional deformation measuring instrument, Digital Image Correlation-3D (DIC-3D), was used to collect strain and crack deformations in concrete and M-section steel. Further, to visualize the cracking process, a layer of putty was painted on the top and sides of the concrete, and randomly distributed scatter spots were created on the concrete and M-section steel surfaces to facilitate the DIC-3D data acquisition. Scattered spots were created, as shown in Figure 6a. Two sets of equipment were used for acquisition to facilitate comparative analysis of the data, as depicted in Figure 6b.

### 2.4. Strain Measurement

**Figure 7:**
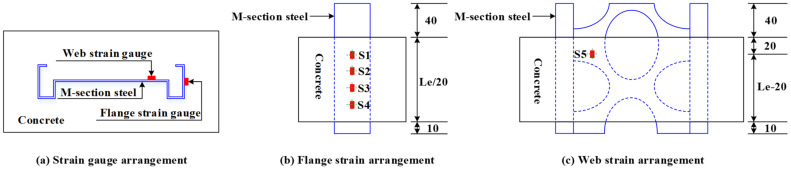
Layout of strain gauges (unit: mm).

To obtain the surface strain distribution pattern of the M-section steel, strain gauges were deployed at the web and flange of the M-section steel, as depicted in Figure 7.

## 3. Experimental Results and Load–Slip Curve of MSSC Specimens

### 3.1. Analysis of Experimental Results

#### 3.1.1. The Failure Pattern of MSSC Specimens

**Figure 8:**
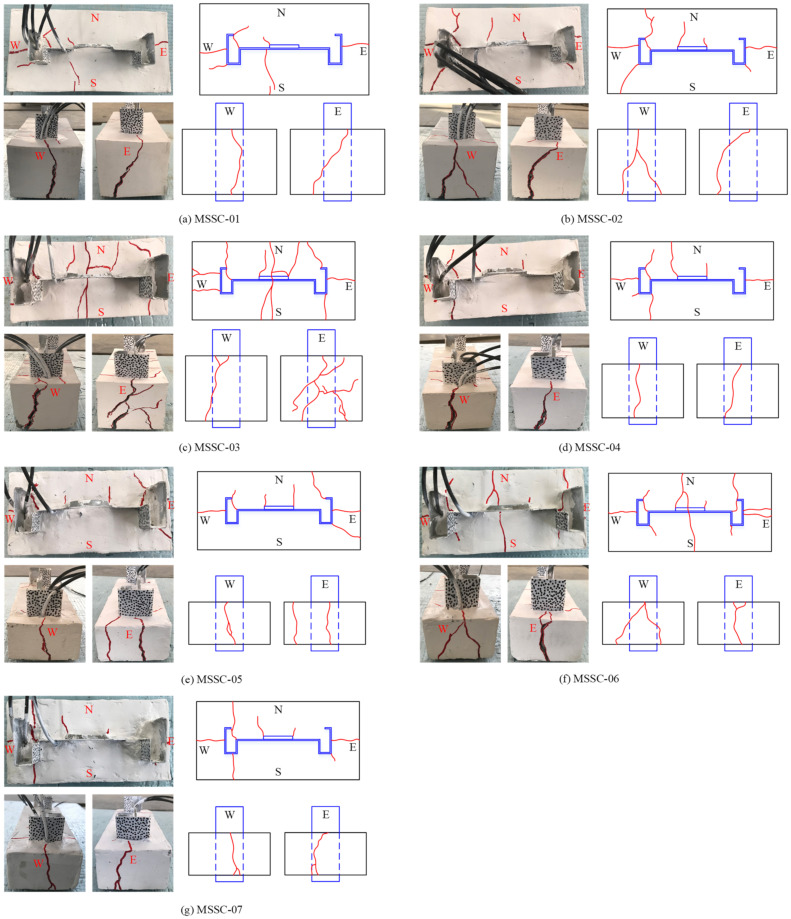
Final failure mode of MSSC specimens.

Figure 8 shows the final failure pattern of each specimen. Owing to a large number of specimens, the failure pattern of one specimen has been presented for each group of specimens, and the remaining two specimens exhibited approximately the same failure pattern. For monotonic loading, owing to the relatively small load at the beginning of the loading, most of the specimens did not exhibit significant slip or microcracks. With an increase in the load, the loading end of the M-section steel gradually slipped slightly, and a small creaking sound inside the specimen indicated the start of the loss of the interfacing chemical adhesive force between the M-section steel and concrete. However, microcracks were not observed on the surface of the specimen.

When loaded to 60–80% of the ultimate load, the specimens exhibited slight vertical cracks along the anchorage length on the east and west sides. However, specimens with thicker concrete covers did not show any cracks, such as MSSC-01 and MSSC-02.

On being subjected to the ultimate load, the specimens produced a large cracking sound, the load suddenly decreased, and the slip of the free end increased significantly. At this point, all the specimens exhibited vertical penetration microcracks in the direction of the anchorage length on the east and west sides, which were located approximately in the middle of the M-section steel flange. Further, vertical microcracks started at the free end and extended to the loading end. Certain specimens also formed new small branching cracks in the direction of the anchorage length on the east and west sides, such as MSSC-02, MSSC-03, MSSC-05, and MSSC-06.

Subsequently, the load decreased significantly. When the load dropped to approximately 20–30% of the ultimate load, cracks in the 45° direction gradually appeared on the concrete surface at the loading end (located at the corners of the M-section steel flanges and the corners of the web reinforcement rolls). Consequently, it exhibited a tendency to extend in all directions. In addition, certain specimens exhibited horizontal cracks along the lateral edges of the M-section steel and web, for example, MSSC-01, MSSC-03, and MSSC-06. However, the vertical microcracks along the length of the anchorage on the east and west sides also expanded outwards. With the increase in slippage of the loading and free ends, the load decreased relatively slowly, and eventually, tended to be horizontal. At this moment, the mechanical bite force and frictional resistance force provided bond strength between the M-section steel and concrete. During this process, cracks developed, and the existing cracks extended rapidly. Most of the specimens exhibited penetrating diagonal cracks on the concrete surface at the loading end. The crack width also gradually increased, and the branch cracks on each side penetrated each other owing to the development of the branch cracks. Finally, it resulted in concrete spalling at the free-end corners of certain specimens. Further, the crack widths were also measured and reached a maximum width of 3–5 mm, with longitudinal splitting cracks predominating.

**Figure 9:**
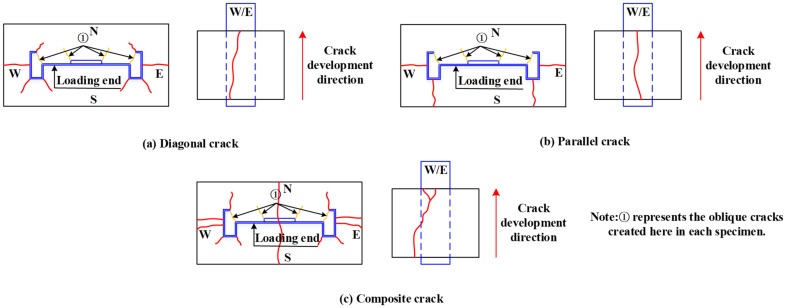
Typical crack pattern.

According to the failure pattern of the specimens, each specimen was found to exhibit oblique cracks at the web reinforcement-roll edge and the corner of the web. This is because of the presence of the web reinforcement-roll edge and holes in the web, which hinder the tendency of the M-section steel to slide downwards. Thus, diagonal tearing failure is generated in the concrete, as shown by the type **①** crack in Figure 9. The type **①** crack started to appear when the load decreased to approximately 20% of the ultimate load and continued to extend outwards. Certain specimens showed cracks extending to their surface. In addition to the type **①** crack, the crack pattern of the specimen can be divided into three categories:

(1) Diagonal crack: First, the specimen developed a through-crack on the east and west sides from the outside towards the inside of the concrete. With the increase in the slip of the loading end, diagonal cracks gradually started to develop on the rolled edges and corners of the connecting section of the M-section steel. They were roughly oriented towards 45° and tended to extend outwards. In the case of certain specimens with a thinner concrete cover, this type of crack penetrated the surface of the specimen. Most specimens exhibited a diagonal crack as the final failure pattern, as illustrated in Figure 9a.

(2) Parallel crack: Similarly, this type of crack first generated a through-crack on the east and west sides of the concrete from the outside towards the inside. Subsequently, a horizontal crack developed in the middle of the M-section steel connecting section from the inside towards the outside, and continued through to the surface of the specimen. In addition, parallel cracking was mainly observed in specimens with a thicker concrete cover and higher concrete strengths, as shown in Figure 9b.

(3) Composite crack: This crack development pattern is complex and is generally a combination of the two crack patterns described above. First, the specimen exhibited two through-cracks on the east and west sides of the concrete from the outside to the inside. Subsequently, an oblique crack in the direction of approximately 45° appeared and extended outwards at the corner of the rolled edge and the connecting section of the M-section steel. In addition, a microcrack developed in the middle of the M-section steel web and extended towards the south and north sides. Finally, with the increase in the slip of the loading end, a roughly vertical through-crack was observed in the middle of the M-section steel web, as depicted in Figure 9c.

#### 3.1.2. Scattering Results of DIC-3D

**Figure 10:**
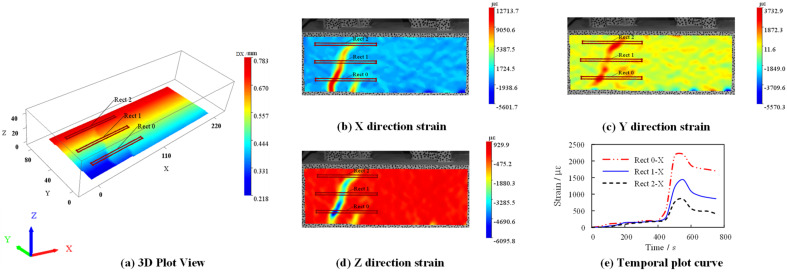
Scattering displacement and strain of MSSC-02 specimen.

Using the DIC-3D scattering technique, Figure 10 shows the displacement and strain variation along the anchorage length on the south side of MSSC-02. It is evident that the variation of the displacement of the specimens in the X-direction was roughly symmetrically distributed, which indicated that the specimens were subjected to uniform forces at the left and right ends. However, owing to the nonuniformity of concrete, the cracks appeared with certain randomness. This resulted in most of the crack development in the specimen not being symmetrically distributed. The strain in the X-direction of the specimen was significantly larger than those in the other two directions; therefore, the specimen was mainly deformed along the X-direction. Nevertheless, there were no obvious cracks in the Y- and Z-directions, which apparently had an obvious restraining effect in the X-direction and prevented crack expansion [27]. As evident from the temporal time plots in Figure 10, the concrete strain started to increase sharply at approximately 400 s for the specimen. However, when the crack developed to a certain form, the strain gradually reached a plateau, indicating that the specimen had entered the residual bond strength stage.

#### 3.1.3. Strain Distribution of M-Section Steel

**Figure 11:**
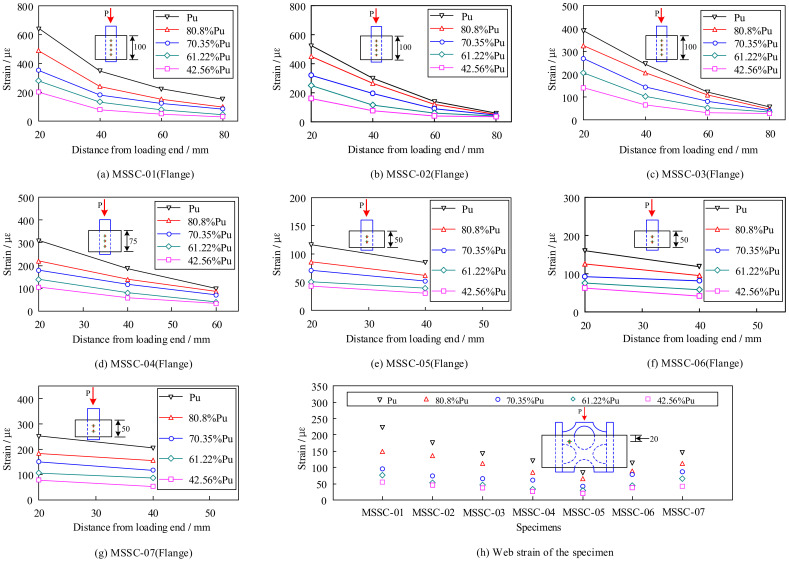
Longitudinal strain distribution at each position of MSSC specimens in the rising section of the load.

To analyze the strain variation on the surface of the M-section steel, it was assumed that the strain was the same at the same horizontal position for the inside and outside of the flange, web, and symmetric positions. Because of the rapid unloading of the specimens after reaching the ultimate load, the strain gauges experienced significant failure. Therefore, Figure 11 shows only the strain curve of each specimen with the position during the rising section of the load. It is evident that the strain in the flange of the specimen varied with position in an exponential function-distribution pattern. The strain near the loading end tended to decrease towards the free end, and the loading end changed more rapidly with the increase in the load. Upon reaching the ultimate load, the maximum strain was 645 με, which was significantly less than the yield strain obtained from the tests for M-section steel (1477 με), thereby indicating that no yielding of the M-section steel occurred during the test. Figure 11h shows the variation in the strain in the web of each specimen 20 mm from the loading end. Owing to the special characteristics of M-section steel, the strain variation in the web occurred at only one location; thus, an analysis of the web strain variation law in terms of the location was not possible. However, when comparing the strains of the flanges at the same position, it can be observed that the strains at the flanges were significantly greater than those at the web, which indicates that the external load was primarily carried by the flanges at the rising section of the load.

### 3.2. Load–Slip Curves and Characteristics

**Figure 12:**
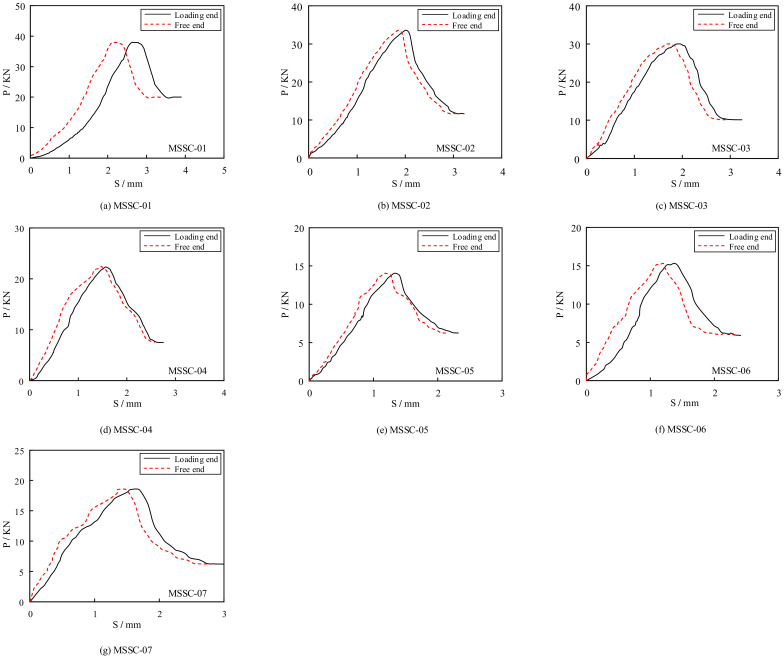
The load–slip curve of MSSC specimens.

**Figure 13:**
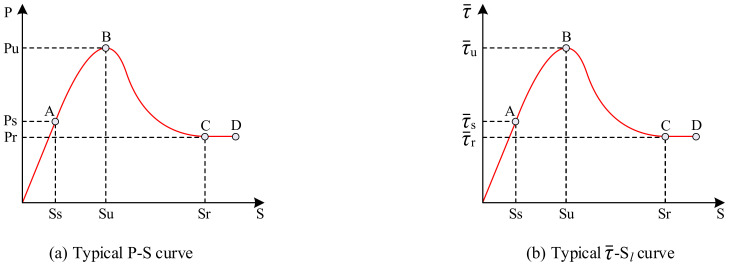
Typical characteristic curves.

To ensure the accuracy of the data, the results of each set of specimens were averaged to obtain the load–slip curve, as shown in Figure 12. It can be observed that although the loading end slips before the free end, the shape of the load–slip curve is generally the same for both. Therefore, the load–slip curve model can be established, as illustrated in Figure 13a, comprising the following four stages:

(1) Linear ascending stage (OA): In the early stages of loading, the specimen initially developed a small slip near the loading end, which caused a gradual reduction in its chemical adhesive force, and the cementitious colloid sheared off. Further, the internally broken concrete layer increased in volume compared with the previous volume of the concrete layer owing to the presence of pores. This resulted in normal stress between the M-section steel and concrete, which generated a frictional resistance force and mechanical bite force. Simultaneously, new chemical adhesive forces developed in the bonding area where no slippage occurred. The newly generated chemical adhesive force, frictional resistance force, and mechanical bite force carried the external load together, which enabled the specimen to bear a stronger external load. During this phase, the slip at the loading end was approximately linearly related to the load. The magnitude of the load corresponding to point A was approximately 40–50% of the ultimate load **Pu**, that is, the initial load **Ps**, which corresponded to the slip value of **Ss**.

(2) Non-linear ascending stage (AB): When the load reached inflection point A, the load–slip curve exhibited a change from the previously linear to a non-linear growth. Within this phase, the chemical adhesive force remained at a constant value in the bonding area where there was no slippage; however, the frictional resistance force and mechanical bite force in the zone where slip occurred were slowed down owing to the continuous cracking and breaking within the concrete. The slip growth rate was faster than the bond growth rate. Consequently, the slope of the curve gradually decreased, and the upward convexity became increasingly obvious. Subsequently, the bonded section without slip was gradually transferred from the loading end to the free end. When the slip reached **Su**, the load reached point B, which was the ultimate load **Pu**. In addition, vertical through-cracks were generated on the east and west sides of the concrete at this point.

(3) Non-linear descending stage (BC): After the load reached its peak, the chemical adhesive force was gradually lost with the continuous increase in the amount of slip. Although the frictional resistance force and mechanical bite force still increased, the increase was much smaller than the loss of the chemical adhesive force. Thus, the load–slip curve exhibited a gradual downward trend. At this stage, the slip had more space to develop as vertical through-cracks developed on the east and west sides of the concrete. However, because of the presence of M-section steel web holes and reinforced rolled edges, normal and shear stresses were generated on the concrete. Consequently, the combined effect of the above-mentioned forces caused diagonal cracks to appear on the surface of the concrete, which continued to extend outwards. Once the slip value reached **Sr**, there was a tendency for the load to level off, that is, the residual load **Pr**, which corresponded to a load size of approximately 30–60% of the ultimate load **Pu**.

(4) Residual stage (CD): As the contact surface of the M-section steel and concrete gradually smoothed, the crack development stabilized, and the frictional resistance and mechanical bite forces tended to attain a constant value. The load–slip curve was approximated to a horizontal line or a slightly decreasing curve (this was because of the further consolidation and crushing of the concrete crystals at the contact surface of the M-section steel and concrete, which resulted in slight degradation of the bond strength between the M-section steel and concrete contact surfaces). With a further increase in the slip, the M-section steel was pushed out. Finally, the specimens were subjected to damage.

To obtain the average bond–slip curve, namely the $\bar{\tau }$-$S_{l}$ curve, the following assumptions must be implemented: the contribution of the M-section steel web openings and the reinforcement-roll edge to the total bonding effect is converted into a natural bonding action. Therefore, the average bond strength can be simplified as the ratio of the magnitude of the load measured using the force transducer and the contact area of the M-section steel to the concrete, as follows:

(1) $$\bar{\tau }=P/\left(C\cdot L_{e}\right)$$

where $\bar{\tau }$ is the average bond strength, P is the external load, C is the perimeter of the cross-section of the M-section steel, and $L_{e}$ is the concrete anchorage length.

According to Equation (1), three corresponding average characteristic bond strength values can be obtained: the average initial bond strength ${\bar{\tau }}_{s}$, average ultimate bond strength ${\bar{\tau }}_{u}$, and average residual bond strength ${\bar{\tau }}_{r}$, respectively; the corresponding slip values are the initial slip value **Ss**, ultimate slip value **Su**, and residual slip value **Sr**, respectively. This curve reflects the basic characteristics of the bond strength of M-section steel to concrete, as shown in Figure 13b. Table 4 lists the specific values for each characteristic point.

## 4. Study on the Bond–Slip Constitutive Model of MSSC

### 4.1. Parametric Analysis of Characteristic Average Bond Strength

#### 4.1.1. Analysis of Influencing Factors on Characteristic Average Bond Strength

**Figure 14:**
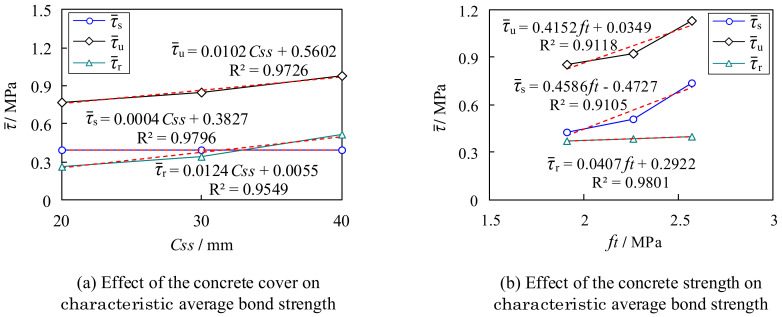

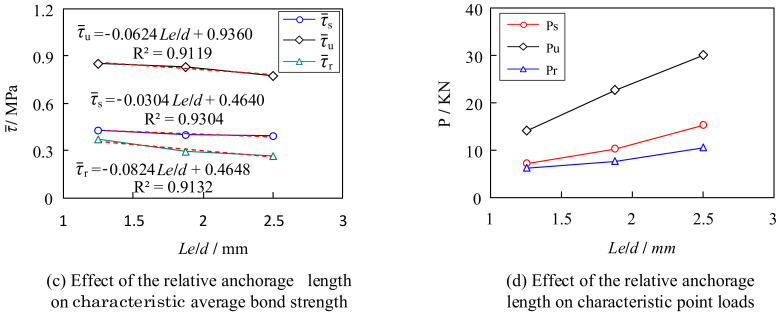
Regression analysis of factors influencing characteristic average bond strength.

To analyze the respective effects of concrete strength, anchorage length, and concrete cover on the bond strength of M-section steel to concrete, a single-factor linear regression analysis was performed on the characteristic average bond strengths derived from the tests, as illustrated in Figure 14.

##### Effect of Concrete Cover on Characteristic Average Bond Strength

Figure 14a presents a diagram of the characteristic average bond strengths ${\bar{\tau }}_{s}$, ${\bar{\tau }}_{u}$, and ${\bar{\tau }}_{r}$ as a function of the concrete cover. As is evident, the average ultimate bond strength ${\bar{\tau }}_{u}$ and the average residual bond strength ${\bar{\tau }}_{r}$ increased roughly linearly with the increase in the thickness of the concrete cover. In particular, the effect on the average ultimate bond strength ${\bar{\tau }}_{u}$ was the most obvious. However, the effect on the average initial bond strength ${\bar{\tau }}_{s}$ was smaller.

##### Effect of Concrete Strength on Characteristic Average Bond Strength

The relationship between the concrete strength and the characteristic average bond strengths is shown in Figure 14b. It is evident that the characteristic average bond strength increased with the increase in the strength of the concrete. This is because the internal cracking, splitting, and extrusion caused by the bond force are all related to the properties of the concrete [37]. However, the effect on the average residual bond strength ${\bar{\tau }}_{r}$ was not significant.

##### Effect of Anchorage Length on Characteristic Average Bond Strength

Figure 14c shows a diagram of the effect of the relative anchorage length $L_{e}/d$ on the characteristic average bond strength, where d denotes the height of the cross section of the M-section steel. It can be deduced that the characteristic average bond strength decreased with the increase in the relative anchorage length. In contrast, the characteristic point load increased, as shown in Figure 14d. This indicates that an increase in the anchorage length improved the overall bonding action of the specimen; however, the bonding action assumed at each point of action for the M-section steel decreased accordingly.

#### 4.1.2. Regression Analysis of Characteristic Average Bond Strength

The statistical regression analysis of the influencing factors revealed that the average initial bond strength ${\bar{\tau }}_{s}$ is mainly influenced by the concrete strength and anchorage length, the average residual bond strength ${\bar{\tau }}_{r}$ is mainly influenced by the concrete cover and anchorage length, and the average ultimate bond strength ${\bar{\tau }}_{u}$ is affected by three factors: concrete cover, concrete strength, and anchorage length. In the following, the influence of the above three factors on the characteristic average bond strength is considered comprehensively. Statistical regression analysis was conducted on the test results to establish the characteristic average bond strength equation, as shown in Equations (2)–(4). In addition, to verify the accuracy of the fitted equation, the calculated results were analyzed and compared with the experimental results, as shown in Table 5. As is evident, the calculated values are in good agreement with the experimental values; this demonstrates the reliability of the equation and shows that the calculation assumptions considered in Section 3.2 are reasonable.

(2) $${\bar{\tau }}_{s}=0.45740f_{t}-0.00720L_{e}/d-0.46100$$

(3) $${\bar{\tau }}_{u}=0.00949C_{ss}+0.39480f_{t}-0.05200L_{e}/d-0.04000$$

(4) $${\bar{\tau }}_{r}=0.01285C_{ss}-0.10900L_{e}/d+0.25940$$

### 4.2. Definition of Characteristic Slip Values and Regression Analysis

To obtain the bond–slip constitutive model of the MSSC, three characteristic slips were also defined according to the experimental $\bar{\tau }$-$S_{l}$ curve; that is, the initial slip value **Ss**, ultimate slip value **Su,** and residual slip value **Sr**. The test results indicated that the initial slip value **Ss** was primarily influenced by the concrete cover and strength of the concrete, in addition to the above two influencing factors, while the ultimate slip value **Su** and residual slip value **Sr** were also influenced by the anchorage length. Based on the analysis above, the regression equation for the characteristic slip values was established as follows:

(5) $$S_{s}=0.01892C_{ss}+0.16620f_{t}+0.08800$$

(6) $$S_{u}=0.03216C_{ss}+0.38800f_{t}+0.40500L_{e}/d-0.49000$$

(7) $$S_{r}=0.03228C_{ss}+0.88800f_{t}+0.56100L_{e}/d-0.98900$$

### 4.3. The Bond–Slip Constitutive Equation of the MSSC

Using the characteristic average bond strength and characteristic slip values defined in the previous section, the $\bar{\tau }$-$S_{l}$ constitutive relationship curve can be divided into four segments, as illustrated in Figure 13b. In this section, the $\bar{\tau }$-$S_{l}$ constitutive relationship is fitted by establishing the corresponding mathematical model for each segment.

(1) Linear ascending stage (OA): At this stage, the average bond strength tended to increase linearly with the amount of slip. Therefore, the description is given in a straight line as follows:

(8) $$\bar{\tau }=aS_{l}+b\;\;(0<S_{l}\leq S_{s})$$

where *a* = ${\bar{\tau }}_{s}$/$S_{s}$, *b* = 0.

(2) Non-linear ascending stage (AB): During this phase, the average bond strength tended to increase non-linearly with the amount of slip. Thus, a hyperbolic curve is used to describe it:

(9) $$\bar{\tau }=\frac{p}{S_{l}}+q\;\;(S_{s}<S_{l}\leq S_{u})$$

where *p* = $\frac{{\bar{\tau }}_{s}-{\bar{\tau }}_{u}}{S_{u}-S_{s}}S_{u}S_{s},$ and *q* = $\frac{{\bar{\tau }}_{u}S_{u}-{\bar{\tau }}_{s}S_{s}}{S_{u}-S_{s}}$.

(3) Non-linear descending stage (BC): During this phase, the average bond strength tended to decrease non-linearly with the amount of slip. Therefore, a hyperbolic curve is used to describe this phase:

(10) $$\bar{\tau }=\frac{m}{S_{l}}+n\;\;(S_{u}<S_{l}\leq S_{r})$$

where *m* = $\frac{{\bar{\tau }}_{u}-{\bar{\tau }}_{r}}{S_{r}-S_{u}}S_{u}S_{r}$, and *n* = $\frac{{\bar{\tau }}_{r}S_{r}-{\bar{\tau }}_{u}S_{u}}{S_{r}-S_{u}}$.

(4) Residual stage (CD): With an increase in slip, the average bond strength remains essentially constant, which can be described by a straight line at this stage:

(11) $$\bar{\tau }={\bar{\tau }}_{r}\;\;\;(S_{l}>S_{r})$$

**Figure 15:**
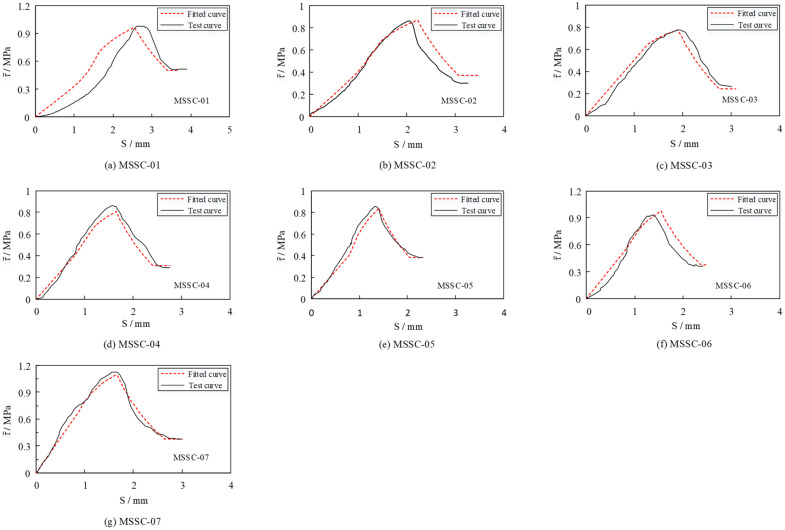
Comparison analysis of the test and fitted curves.

To examine the degree of curve fitting, Figure 15 provides a comparative analysis of the test and fitted curves. It is evident that the fitted curve can reflect the shape and characteristics of the test curve more realistically, which demonstrates the reasonableness of the bond–slip constitutive equation.

## 5. Finite Element Verification of the Bond–Slip Constitutive Model of MSSC

### 5.1. General

**Figure 16:**
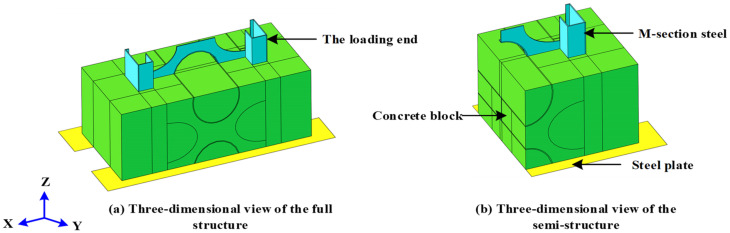
Finite element model.

To further investigate the reasonableness of the proposed constitutive relationship, this section presents additional validations performed by simulating a push-out test using the finite element software ABAQUS 2020. Owing to the complexity of the internal bonding mechanism of the MSSC specimen, convergence with the standard solution is difficult. Therefore, a quasi-static analysis of 21 MSSC specimens was conducted using the explicit dynamic solution method. Further, to ensure the accuracy of the results, the overall kinetic energy of the specimen must be maintained below 10% of its internal energy [38]. Exploiting the symmetry of this structure, a semi-structure was built to simplify the model. Consequently, the basic part of the finite element model comprised M-section steel, a concrete block, and a steel plate, as shown in Figure 16.

### 5.2. Finite Element Type

**Figure 17:**
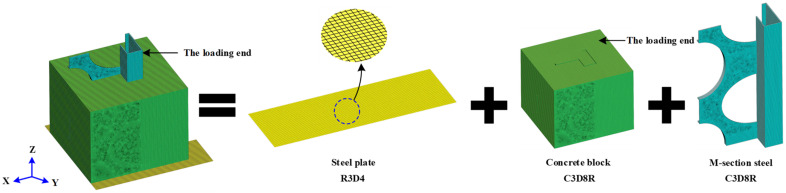
Finite element type and mesh.

The element type and meshing of the finite element model are presented in Figure 17, where the concrete block and M-section steel both used an eight-node linear hexahedral reduced integral element (C3D8R) [22], whereas the steel plate used a discrete rigid-body element (R3D4) [15].

### 5.3. Interaction

**Figure 18:**
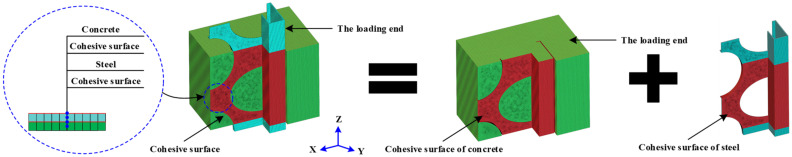
Contact details of the FE model.

To define the interaction of the concrete with M-section steel and concrete-to-steel plates, the general contact provided in ABAQUS was used. The details of the finite-element contact between the M-section steel and concrete are illustrated in Figure 18. The contact surface of the M-section steel acted as the master surface, whereas the concrete surface acted as the slave surface. In a similar manner, for the contact surface between the concrete and steel plate, the surface of the steel plate was used as the master surface, while the concrete surface was used as the slave surface. Further, the normal contact behavior used hard contact, thereby allowing pressure to be transferred between surfaces. In contrast, the tangential contact behavior used a penalty-friction formulation with a friction coefficient of 0.5 [39,40]. In addition, the cohesive damage unit was used to specify the bond–slip between the contact surface of the M-section steel and the concrete, and its cohesive properties are detailed in Section 5.6.

### 5.4. Boundary Conditions and Loading Methods

**Figure 19:**
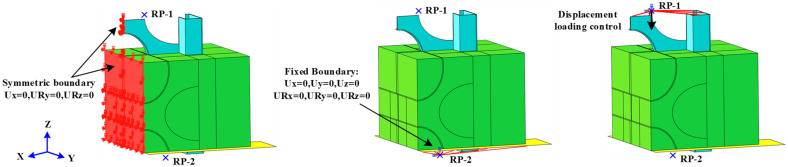
Boundary conditions and loading methods.

Owing to the symmetry of the specimen in the YZ plane, all the nodes on the symmetry plane of the MSSC specimen are limited in terms of translation in the X-direction and rotation in the Y- and Z-directions, as shown in Figure 19. A reference point, RP-2, was established to constrain the steel-plate coupling to a single point and limit its translation and rotation in three directions, which was used to simulate the bottom boundary conditions in the test. Moreover, to prevent errors due to eccentricity, the top surface on the loading end of the M-section steel was coupled to RP-1, which was loaded via displacement control.

### 5.5. Material Properties

During the FEA, a plastic-damage model (CDP) was used for concrete. This damage model is based on a plastic continuous medium and assumes tensile cracking and compressional crushing of the concrete material as its main failure mechanisms, with equivalent tensile plastic strain and equivalent compressive plastic strain controlling the evolution of the failure surface [41]. The basic parameters of the CDP model are listed in Table 6, where **Ψ** is the dilation angle, ∈ refers to the eccentricity, $f_{b0}/f_{c0}$ is the ratio of the initial equibiaxial compressive yield stress to the initial uniaxial compressive yield stress, and **Kc** is the ratio of the second stress invariant to the tensile meridian [42]. The uniaxial tensile and compressive stress–strain curves for concrete were obtained using the data acquired from the tests, and the specific parameters of the material are detailed in Section 2.2. In addition, referring to the ABAQUS/Explicit 6.14 user manual, the mechanical behavior of the tensile cracking and compression breaking of concrete was described by introducing damage factors, as shown in Equations (12) and (13).

(12) $$\sigma_{c}=\left(1-d_{c}\right)E_{0}\left(\varepsilon_{c}-\varepsilon_{c}^{pl}\right)$$

(13) $$\sigma_{t}=\left(1-d_{t}\right)E_{0}\left(\varepsilon_{t}-\varepsilon_{t}^{pl}\right)$$

where $E_{0}$ is the initial (undamaged) modulus of the concrete, $d_{c}$ and $d_{t}$ are the uniaxial damage variables for compression and tension, respectively, and $\varepsilon_{c}^{pl}$ and $\varepsilon_{t}^{pl}$ are the compressive and tensile equivalent plastic strains, respectively [38].

A bilinear strengthening model was used for M-section steel [43]. It was assumed that the tensile and compressive stress–strain curves of the steel were identical, and the basic parameters were obtained from the M-section steel tensile test, as detailed in Section 2.2.

### 5.6. Cohesive Properties

In the finite element program ABAQUS, two modeling methods are provided for the interaction behavior of interfaces, namely cohesive-element and cohesive-contact [38]. The cohesive-contact method was used to define the cohesive interfacial properties between the M-section steel and concrete in this study. The cohesive-contact approach employs a traction–separation law criterion to obtain possible damage patterns at the interface using shear and tensile damage criteria [41]. When combined with the experimental bond–slip curves, a bilinear mixed-mode softening criterion can better describe the interface response between the M-section steel and concrete. Here, $\sigma_{I}$ and $\delta_{I}$ denote the stresses and relative displacements in the pure normal mode, respectively, and $\sigma_{\mathrm{II}}$ and $\delta_{\mathrm{II}}$ indicate the stresses and relative displacements in the pure shear mode, respectively. The specific expressions are expressed as Equations (14) and (15). The relative displacement in the mixed-mode is defined as shown in Equation (16).

(14) $$\delta_{I}=\max\left(\delta_{1},0\right)$$

(15) $$\delta_{\mathrm{II}}=\sqrt{\delta_{2}^{2}+\delta_{3}^{2}}$$

(16) $$\delta_{m}=\sqrt{\delta_{I}^{2}+\delta_{\mathrm{II}}^{2}}$$

where $\delta_{1}$ represents the normal phase of the relative displacement at the interface, and $\delta_{2}$ and $\delta_{3}$ represent the tangential phase of the relative displacement at the interface. The quadratic separation criterion was used to describe the initiation of interface damage, as shown in Equation (17), and its corresponding damage-initiation displacement $\delta_{m}^{e}$ is expressed as Equation (18).

(17) $$\sqrt{{\left(\frac{\max\left(\sigma_{I},0\right)}{\sigma_{I}^{max}}\right)}^{2}+{\left(\frac{\sigma_{\mathrm{II}}}{\sigma_{\mathrm{II}}^{max}}\right)}^{2}}=1$$

(18) $$\delta_{m}^{e}=1/\sqrt{{\left(K_{I}cosI/\sigma_{I}^{max}\right)}^{2}+{\left(K_{\mathrm{II}}cos\mathrm{II}/\sigma_{\mathrm{II}}^{max}\right)}^{2}}$$

(19) $$\cos I=\delta_{I}/\delta_{m}$$

(20) $$\cos\mathrm{II}=\delta_{\mathrm{II}}/\delta_{m}$$

(21) $$K_{I}=\sigma_{I}^{\max}/\delta_{I}^{\max}$$

(22) $$K_{\mathrm{II}}=\sigma_{\mathrm{II}}^{\max}/\delta_{\mathrm{II}}^{\max}$$

where $\sigma_{I}^{\max}$ and $\sigma_{\mathrm{II}}^{\max}$ represent the peak stresses in the pure normal and pure shear modes, respectively, and $\delta_{I}^{\max}$ and $\delta_{\mathrm{II}}^{\max}$ indicate the relative displacements corresponding to the peak stresses in the pure normal and pure shear modes, respectively. Further, ***cos I*** and ***cos II*** are directional cosines, as shown in Equations (19) and (20), $\delta_{m}$ indicates the effective separation displacement value in the mixed-mode during loading, and $\delta_{I}$ and $\delta_{\mathrm{II}}$ indicate the relative displacement values corresponding to the effective separation displacement values in the pure normal mode and pure shear mode surfaces, respectively, during the mixed-mode. In addition, $K_{I}$ and $K_{\mathrm{II}}$ denote the interfacial normal and shear stiffnesses, respectively, and are expressed as Equations (21) and (22).

For damage evolution, the Benzeggagh-Kenane (B-K) form based on energy is used [38], with the specific expression expressed as Equation (23).

(23) $${\left(\frac{G_{I}}{G_{IC}}\right)}^{\alpha }+{\left(\frac{G_{\mathrm{II}}}{G_{\mathrm{II}C}}\right)}^{\alpha }=1$$

(24) $$G_{I}=1/2\sigma_{I}^{Y}\delta_{m}^{f}\cos I$$

(25) $$G_{\mathrm{II}}=1/2\sigma_{\mathrm{II}}^{Y}\delta_{m}^{f}\cos\mathrm{II}$$

(26) $$G_{IC}=1/2\sigma_{I}^{\max}\delta_{I}^{f}$$

(27) $$G_{\mathrm{II}C}=1/2\sigma_{\mathrm{II}}^{\max}\delta_{\mathrm{II}}^{f}$$

(28) $$\sigma_{I}^{Y}=K_{I}\delta_{m}^{e}\cos I$$

(29) $$\sigma_{\mathrm{II}}^{Y}=K_{\mathrm{II}}\delta_{m}^{e}\cos\mathrm{II}$$

where $G_{I}$ and $G_{\mathrm{II}}$ indicate the critical energy release rates of the mixed-mode corresponding to the energy release rates on the pure normal and pure shear mode surfaces, respectively. The specific expressions are shown in Equations (24) and (25). $G_{IC}$ and $G_{\mathrm{II}C}$ represent the critical energy release rates in the pure normal and pure shear modes, respectively, as shown in Equations (26) and (27). Further, $\delta_{I}^{f}$ and $\delta_{\mathrm{II}}^{f}$ denote the relative displacement values of the interface failure in pure normal and pure shear modes, respectively. In addition, $\sigma_{I}^{Y}$ and $\sigma_{\mathrm{II}}^{Y}$ indicate the yield stresses corresponding to the damage-initiation point in the pure normal mode and pure shear mode surfaces during the mixed-mode, respectively, and are expressed in Equations (28) and (29). Furthermore, $\delta_{m}^{f}$ represents the relative displacement corresponding to the complete separation of the interface in the mixed-mode and **α** indicates the B-K parameter.

The degradation of the interface stiffness was described by introducing damage parameter **D**, which is expressed as follows:

(30) $$D=\frac{\sigma_{m}^{f}\left(\delta_{m}-\delta_{m}^{e}\right)}{\delta_{m}\left(\sigma_{m}^{f}-\delta_{m}^{e}\right)}$$

In summary, there are only seven unknown parameters in the above equation: $\delta_{I}^{f}$, $\delta_{\mathrm{II}}^{f}$, $\sigma_{I}^{\max}$, $\sigma_{\mathrm{II}}^{\max}$, $\delta_{I}^{\max}$, $\delta_{\mathrm{II}}^{\max}$, and **α**. In this study, according to the obtained bond–slip curve and the parametric analysis of the finite element model, setting $\sigma_{I}^{\max}$ **=** $\sigma_{\mathrm{II}}^{\max}$ **=** ${\bar{\tau }}_{u}$, $\delta_{I}^{\max}$ **=** $\delta_{\mathrm{II}}^{\max}$ **=** $S_{u}$, $\delta_{I}^{f}$ **=** $\delta_{\mathrm{II}}^{f}$ **=** $S_{r}$, and **α** = 2 [44,45] can better simulate the test results.

### 5.7. Results of Finite Element Constitutive Model Validation

#### 5.7.1. Failure Mode

**Figure 20:**
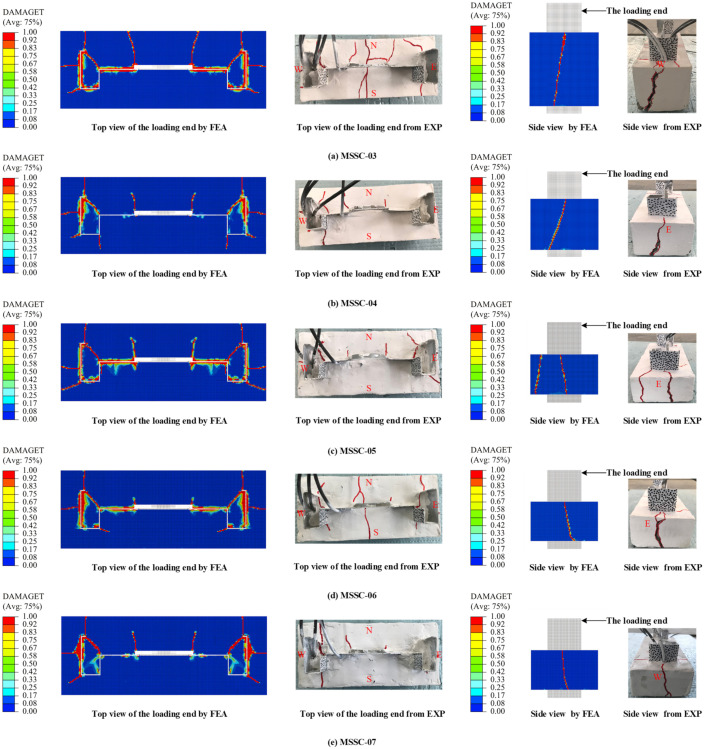
Comparison of FEA and EXP crack development.

Figure 20 presents a comparison between the finite element and test crack development, and the results of the FEA are consistent with the experimental (EXP) phenomenon. The specimen started with a vertical through-crack that extended from the free end towards the loading end on the east and west sides of the concrete. With the increase in the load, diagonal cracks gradually appeared outwards at the corners of the M-section steel flange and web. In reality, concrete is a non-uniform material, while the structural material is assumed to be uniform and symmetrically distributed in the FEA, which results in partial through-cracks that do not appear in the FEA.

#### 5.7.2. Bond–Slip Curve

**Figure 21:**
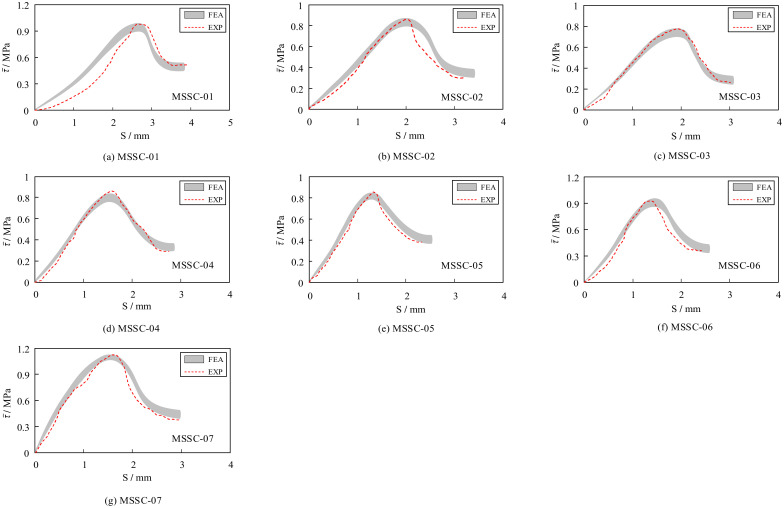
Comparison between FEA and EXP bond–slip curves.

The results of the FEA of 21 MSSC specimens were calculated and compiled to obtain a comparative analysis of the FEA and EXP bond–slip curves, as shown in Figure 21. It is evident that the bond–slip curve derived from the FEA is also roughly divided into four stages: the linear ascending, non-linear ascending, non-linear descending, and residual stages, respectively. The consistency with the EXP results indicates the reasonableness of the proposed bond–slip constitutive relationship.

#### 5.7.3. Stress Clouds of MSSC Specimens

**Figure 22:**
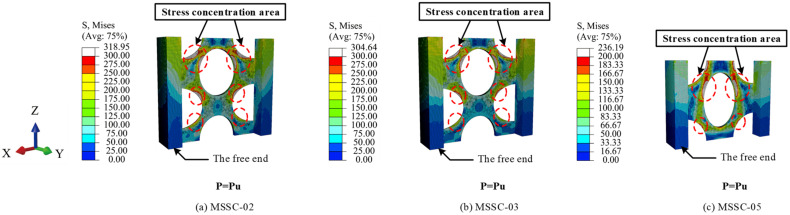
Stress cloud diagram of M-section steel.

Figure 22 shows the stress clouds of the M-section steel when the load reached P = Pu. For the specimens of MSSC-02, MSSC-03, and MSSC-5, the stress variations exhibited the same development pattern. Thereafter, with the increase in the slip, the stress concentrations in the M-section steel first appeared at the upper edge of the central through-hole and the upper and lower edges of the curved through-hole, indicating a strong interaction between them. However, this resulted in a tendency to resist shear, thus stopping the tendency of the M-section steel to slide downwards. Considering specimen MSSC-03 as an example, it was smashed open to allow us to observe the internal conditions. The concrete inside the central through-hole and the curved through-hole were found to be significantly loose. Further, the concrete at the upper edge of the central through-hole and the upper and lower edges of the curved through-hole were significantly looser than that in the other parts.

**Figure 23:**
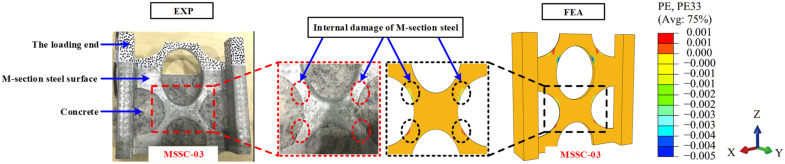
Internal failure mode comparison of M-section steel for specimen MSSC-03.

However, the M-section steel did not buckle, and the FEA results confirmed that the M-section steel entered a plastic state, as illustrated in Figure 23.

#### 5.7.4. Interfacial Bond-Failure Process

**Figure 24:**
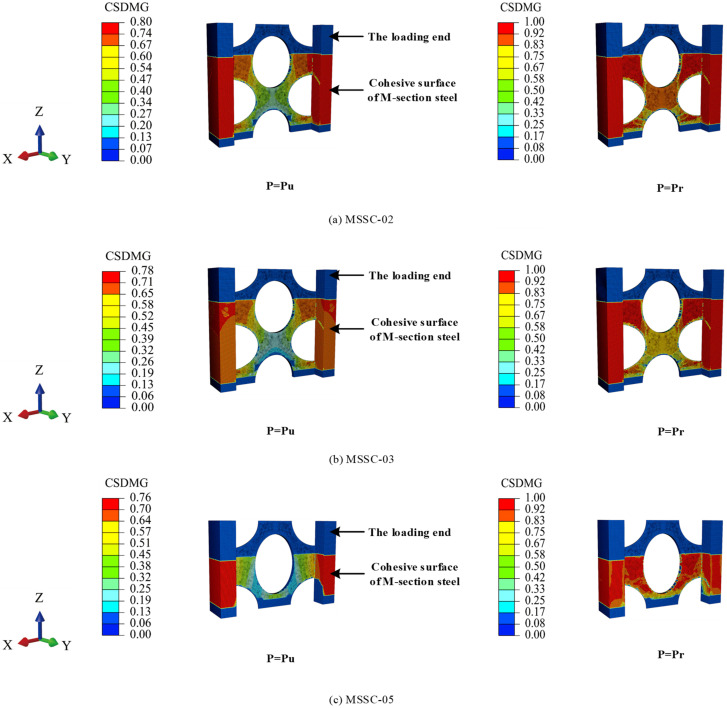
The process of MSSC specimen interface failure.

**Figure 25:**
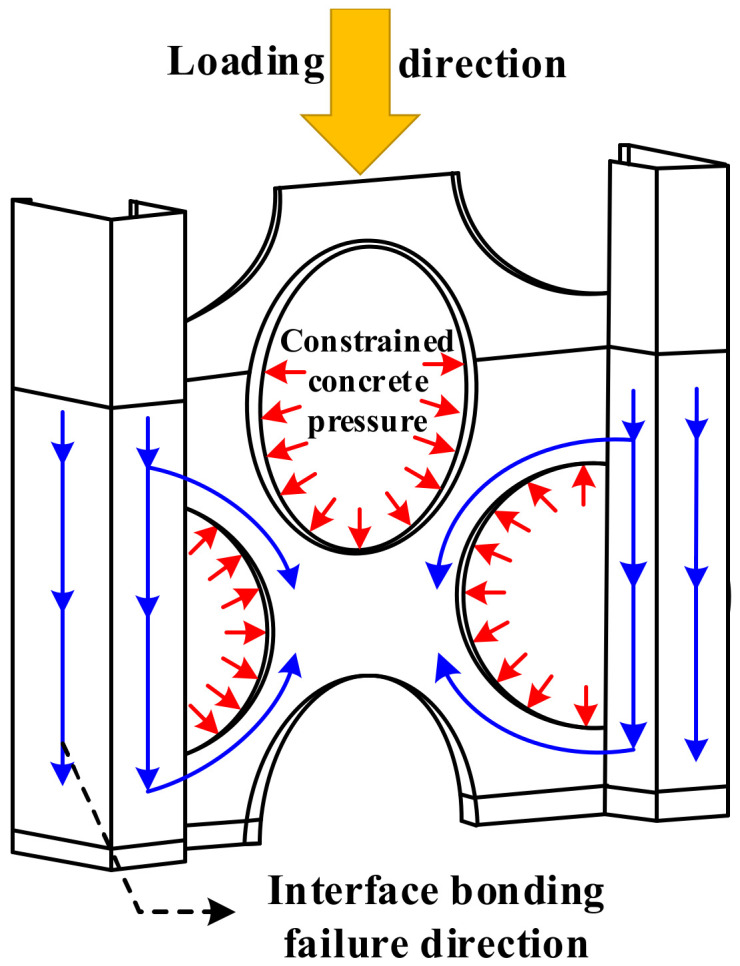
Sketch of interface bonding-failure effect.

Figure 24 shows the process of interfacial bond damage in a part of the specimen. At the beginning of loading, no damage to the interface between the M-section steel and concrete was observed. However, with the increase in the load, interface damage started to appear in the direction of the loading end of the M-section steel flange and web and continued to extend towards the loading direction. Further, when the M-section steel flange interface damage extended to the lower edge of the curved through-hole (at which point, the web interface damage also slowly extended in the direction of loading), the web interface damage also began to extend in the opposite direction to loading because of the internally restrained concrete preventing the M-section steel from sliding downwards, as shown in Figure 25. Thereafter, the load reached a peak Pu when the area of interfacial damage in the center of the web was penetrated. At this point, the interfacial damage was approximately 0.8, as exhibited in specimens MSSC-02 and MSSC-03 in Figure 24 (where specimen MSSC-05 also exhibits the same development pattern). With the increase in the slip of the M-section steel, the damage at the interface between the center of the web and the flange intensified. Until the interface damage reached 1, the M-section steel was completely separated from the concrete part of the interface area, where the load reached the residual load Pr. Furthermore, the FEA results indicated that this interfacial bond-failure process roughly reflected the cracking process of concrete during the test.

## 6. Conclusions

In this study, push-out tests were conducted on 21 MSSC specimens to investigate the effects of the concrete cover, concrete strength, and anchorage length on the bond strength of the M-section steel to concrete. The failure patterns of the MSSC specimens were analyzed, and the displacement and strain variation states of the specimens were investigated using a non-contact optical three-dimensional deformation measuring instrument, DIC-3D. Thereafter, the bond–slip constitutive equation for M-section steel to concrete was proposed. Further, to verify the reasonableness of the constitutive equation, a comparative analysis of the experimental results was performed. In addition, to reproduce the entire failure process of the MSSC specimen, the proposed constitutive equations were simulated numerically. Based on the results of the experiments and numerical simulations, the following conclusions were drawn.

- Depending on the final failure pattern of the MSSC specimen, the crack pattern could be divided into three main types: diagonal, parallel, and composite cracks. The diagonal crack occurred mainly in specimens with a thinner concrete cover and was the predominant crack pattern. Parallel cracks were mainly observed in the specimens with a greater concrete cover thickness and higher concrete strength. However, the composite crack development pattern was more complex and occurred only in a few specimens.
- The DIC-3D scattering results showed that the displacement variation of the MSSC specimen in the X-direction was symmetrically distributed, and the specimen was mainly deformed in the X-direction, whereas the Y-direction and Z-direction had an obvious restraining effect on deformation in the X-direction.
- The load–slip curve of the MSSC specimen was divided into four major stages: the linear ascending, non-linear ascending, non-linear descending, and residual stages. In the early stages of loading, the specimen initially slipped slightly near the loading end where the bond strength was mainly supplied by the chemical adhesive force, but no cracks were produced. With the increase in the amount of slip, cracks gradually emerged on the concrete surface, and the load reached the ultimate peak Pu when vertical through-cracks appeared on the concrete sides. With a further increase in slip, the chemical adhesive force was gradually lost, while the frictional resistance and mechanical bite forces gradually increased. However, the growth was much less than the loss of chemical adhesive force, which resulted in a non-linear decrease in the load and the amount of slip. Consequently, when the load decreased to a certain value, it ceased to grow, reaching the residual load Pr. Here, the bond strength was mainly provided by a combination of frictional resistance and mechanical bite forces.
- Through a linear regression analysis of the test results, it was observed that the initial average bond strength ${\bar{\tau }}_{s}$ was mainly influenced by the concrete strength and anchorage length, while the residual average bond strength ${\bar{\tau }}_{r}$ was mainly affected by the concrete cover and anchorage length. In contrast, the ultimate average bond strength ${\bar{\tau }}_{u}$ was influenced by the concrete strength, concrete cover, and anchorage length. Based on this, the characteristic average bond strength equation was established, and a statistical regression method was used to obtain the bond–slip constitutive equation for M-section steel to concrete. Furthermore, a comparison with the test curves was verified, and the results showed that the fitted curves were consistent with the test curves.
- To further validate the reasonableness of the proposed bond–slip constitutive equation of M-section steel to concrete, numerical simulations were performed on the MSSC specimens. The results showed that the numerical simulation can better predict the entire failure process of the specimen. Moreover, the bond–slip curves obtained through the numerical simulation were consistent with the test results, thereby indicating the reliability of the constitutive relationship.

## Figures and Tables

**Table 1:** Specimen parameters.

Specimen	Concrete Cover, Css/mm	Design Strength $f_{cu}$/MPa	Anchorage Length, $L_{e}$/mm	Number
MSSC-01	40	20	100	3
MSSC-02	30	20	100	3
MSSC-03	20	20	100	3
MSSC-04	20	20	75	3
MSSC-05	20	20	50	3
MSSC-06	20	25	50	3
MSSC-07	20	30	50	3

**Table 2:** Properties of concrete materials.

Design Strength	Modulus of Elasticity, Ec/MPa	Average Compressive Strength, $f_{cu}$/MPa	Average Tensile Strength, $f_{t}$/MPa
C20	24,325	19.92	1.91
C25	27,624	25.63	2.26
C30	29,536	31.01	2.57

**Table 3:** Material properties of the steel.

Steel Type	Young’s Modulus, E/MPa	Yield Stress, $f_{y}$/MPa	Ultimate Stress, $f_{u}$/MPa	Elongation, A/%
M-section steel	202,000	298.29	375.33	27

**Table 4:** List of feature point data.

Specimen	$P_{s}$/KN	${\bar{\tau }}_{s}$/MPa	$P_{u}$/KN	${\bar{\tau }}_{u}$/MPa	$P_{r}$/KN	${\bar{\tau }}_{r}$/MPa	$S_{s}$/mm	$S_{u}$/mm	$S_{r}$/mm
MSSC-01	15.503	0.399	37.937	0.976	20.032	0.515	1.198	2.623	3.560
MSSC-02	15.328	0.394	33.575	0.845	13.751	0.345	0.901	2.084	2.953
MSSC-03	15.192	0.391	30.082	0.773	10.433	0.268	0.794	1.911	2.760
MSSC-04	10.336	0.401	22.593	0.833	7.678	0.292	0.796	1.792	2.514
MSSC-05	7.054	0.429	14.032	0.851	6.131	0.371	0.801	1.363	2.050
MSSC-06	8.312	0.509	15.038	0.924	6.204	0.382	0.833	1.471	2.251
MSSC-07	12.081	0.735	18.562	1.128	6.422	0.398	0.898	1.692	2.698

**Table 5:** Comparison of test and calculated values on characteristic average bond strength.

Specimen	Initial Bond Strength, ${\bar{\tau }}_{s}$/MPa		Calculated/Test	Ultimate Bond Strength, ${\bar{\tau }}_{u}$/MPa		Calculated/Test	Residual Bond Strength, ${\bar{\tau }}_{r}$/MPa		Calculated/Test
	Test	Calculated		Test	Calculated		Test	Calculated	
MSSC-01	0.399	0.395	0.989	0.976	0.964	0.987	0.515	0.501	0.973
MSSC-02	0.394	0.395	1.002	0.845	0.869	1.028	0.345	0.372	1.079
MSSC-03	0.391	0.395	1.009	0.773	0.774	1.001	0.268	0.244	0.910
MSSC-04	0.401	0.399	0.995	0.833	0.806	0.968	0.292	0.312	1.069
MSSC-05	0.429	0.404	0.941	0.851	0.839	0.986	0.371	0.380	1.025
MSSC-06	0.509	0.564	1.108	0.924	0.977	1.057	0.382	0.380	0.995
MSSC-07	0.735	0.706	0.960	1.128	1.099	0.975	0.398	0.380	0.955

**Table 6:** The CDP model parameters for concrete.

Dilation Angle (Ψ)	Eccentricity (∈)	$f_{b0}/f_{c0}$	$K_{c}$	Viscosity Parameter
30°	0.1	1.16	0.6667	0.005

## Data Availability

Not applicable.
